# Evolutionary analysis of *Mycobacterium bovis* genotypes across Africa suggests co-evolution with livestock and humans

**DOI:** 10.1371/journal.pntd.0008081

**Published:** 2020-03-02

**Authors:** Osvaldo Frederico Inlamea, Pedro Soares, Cassia Yumi Ikuta, Marcos Bryan Heinemann, Sara Juma Achá, Adelina Machado, José Soares Ferreira Neto, Margarida Correia-Neves, Teresa Rito

**Affiliations:** 1 Programa de pós-graduação Ciência Para o desenvolvimento (PGCD)–Instituto Gulbenkian de Ciência–Portugal; 2 Faculdade de Medicina Veterinária e Zootecnia (VPS-FMVZ), Universidade de São Paulo, USP–Brasil; 3 Instituto Nacional de Saúde, Ministério de Saúde, Moçambique; 4 Faculdade de Veterinária (FAVET), Universidade Eduardo Mondlane, Maputo, Moçambique; 5 Centre of Molecular and Environmental Biology (CBMA), School of Sciences, University of Minho, Braga, Portugal; 6 Institute of Science and Innovation for Bio-Sustainability (IB-S), University of Minho, Braga, Portugal; 7 Direcção de Ciências Animais, Instituto de Investigação Agrária de Moçambique, Ministério de Agricultura e Segurança Alimentar, Maputo, Moçambique; 8 Life and Health Sciences Research Institute (ICVS), School of Medicine, University of Minho, Braga, Portugal; 9 ICVS/3B's, PT Government Associate Laboratory, Braga/Guimarães, Portugal; Beijing Institute of Microbiology and Epidemiology, CHINA

## Abstract

*Mycobacterium bovis* is the pathogenic agent responsible for bovine tuberculosis (bTB), a zoonotic disease affecting mostly cattle, but also transmittable to humans and wildlife. Genetic studies on *M*. *bovis* allow to detect possible routes of bTB transmission and the identification of genetic reservoirs that may provide an essential framework for public health action.

We used a database with 1235 *M*. *bovis* genotypes collected from different regions in Africa with 45 new Mozambican samples. Our analyses, based on phylogeographic and population genetics’ approaches, allowed to identify two clear trends. First, the genetic diversity of *M*. *bovis* is geographically clustered across the continent, with the only incidences of long-distance sharing of genotypes, between South Africa and Algeria, likely due to recent European introductions. Second, there is a broad gradient of diversity from Northern to Southern Africa with a diversity focus on the proximity to the Near East, where M. bovis likely emerged with animal domestication in the last 10,000 years.

Diversity indices are higher in Eastern Africa, followed successively by Northern, Central, Southern and Western Africa, roughly correlating with the regional archaeological records of introduction of animal domesticates. Given this scenario *M*. *bovis* in Africa was probably established millennia ago following a concomitant spread with cattle, sheep and goat. Such scenario could translate into long-term locally adapted lineages across Africa.

This work describes a novel scenario for the spread of *M*. *bovis* in Africa using the available genetic data, opening the field to further studies using higher resolution genomic data.

## Author summary

We describe the genetic diversity distribution in Africa of the pathogen *Mycobacterium bovis*, the responsible for bovine tuberculosis, mostly present in cattle but also transmittable to other animals including humans. This diversity is geographically clustered within the African continent meaning that the genetic diversity was established through independent evolution within different areas. Higher diversity values of *M*. *bovis* are found in Eastern and Northern Africa, followed by Central Africa, with Western and Southern Africa displaying the lowest diversity. These levels of diversity correlate well with the introduction of domesticated livestock in the different regions of Africa, following their domestication in the Near East 10,000 ago. We hypothesize that *M*. *bovis* emerged in the Near East and it was carried across Africa together with domesticated animals and people that developed herding practices and biological tolerance for digesting milk in adulthood. Such scenario implies a strong evolution and co-evolution of *M*. *bovis* across Africa leading to locally adapted strains that could prove a challenge for public health actions.

## Introduction

*Mycobacterium bovis* is the major causal agent of bovine tuberculosis (bTB) in cattle, the world's most neglected zoonotic disease that needs urgent attention, especially in developing countries [1, 2]. The largest disease burden probably occurs in poor, marginalized, rural communities living in close proximity to animals, and with reduced access to safe food and health care. bTB in humans is associated with consumption of unpasteurized dairy products or raw meat products from infected cattle [3–5], or even through inhalation of bacilli when in close proximity to infected cattle or their carcasses [3].

In 2017, Olea-Popelka and co-authors raised awareness for the worldwide importance of bTB, particularly in the African continent, where insufficient surveillance, testing and traditional ways of rearing and consuming cattle may be responsible for under-notification of this zoonotic disease [6]. In fact, an important percentage of cases diagnosed as TB and attributed to *Mycobacterium tuberculosis* infection may be bTB. The disease also impacts on international trade of animals and animal products [7]. The movement of animals has most probably been the main reason for the spread of *M*. *bovis* both within the same country as well as cross-borders [4, 8, 9].

Little is known about the prevalence and epidemiology of bTB in Africa, and its impact on humans, on livestock and wildlife. In sub-Saharan Africa, few studies describe the transmission status of bTB in regions where multiple hosts are present, but it is believed that sharing of water points plays a key role in the transmission and spread of the disease between animals [10]. Indeed, the distribution and epidemiology of bTB varies according to the production system, however, importance in public health and control requires attention of all producers regardless of the system [11].

Different studies have shown that the distribution of bTB in Africa is not uniform, with areas of low and high prevalence [4, 12]. However, It is estimated that 85% of the bovine population and 82% of the human population live in areas where bTB is prevalent and control measures are non-existent or inadequate and the potential for zoonotic transmission is exacerbated by direct coexistence between the breeding/shepherds and cattle [3, 13]. bTB is present in most countries [3, 7] and the political borders do not constitute physical barriers to the movement of animals and their diseases.

In Mozambique, this scenario is common and bTB is assumed to be an important cause of the economic losses in bovine production evidenced by the rejection of carcasses at the slaughterhouse and limitations on national and international trade. However, little is known about the impact of bTB on humans. Mozambique is one of the countries most affected by the TB, TB / HIV and MDR-TB triad with six other African countries, two of which are countries that share borders with Mozambique [14].

The transport of animals might have been a major determinant for the spread of the disease since pre-historic periods. The genetic coalescence of *M*. *bovis* have been estimated to at least around 6000 years [15] and domesticated cattle existed along humans in Africa for at least 8–6000 years [16]. The so-called Secondary Product Revolution involved the continuous use of animal products as milk and wool, likely leading to an increase of animals living closely together in herds. Milk consumption left a major mark on human biology and the genetic acquiring of lactose intolerance in human adulthood is arguably one of the clearest signals of positive selection in populations from Europe and Africa [17–19].

Genetic studies on *M*. *bovis* allow the possibility to detect possible routes of bTB transmission within and across borders. The main objective of this work is to shed light on the presence of genetic reservoirs of bTB in Africa and to trace historical/prehistoric events in the continent using genetic diversity and phylogeography of *M*. *bovis* in Africa.

## Methods

### Ethics statement

Institutional permission to conduct the study was obtained from the National Directorate of Veterinary Services in Maputo, Mozambique (Nota 162/ MINAG/DNSV/900/2013) and Ethical Principles in Animal Research adopted by Ethic Committee in the Use of Animals (CEUA) of School of Veterinary Medicine and Animal Science, University of São Paulo (Protocol number CEUAx 6755081216). Sampling and culling were performed as part of the Veterinary Services regular activity for disease control, following the procedures determined by the Mozambican Animal Health Regulation (Regulamento de Sanidade Animal decreto 26/2009). The slaughter was done in registered abattoirs according to stipulated procedures. All mycobacterial cultures were performed in the National Tuberculosis Reference Laboratory, Ministry of Health Mozambique.

### *Mycobacterium bovis’* samples from Mozambican cattle: collection and genotyping

Forty five samples, consisting of tissue/organs containing lesions, were processed in the BSL3 from the National Reference Laboratory of Tuberculosis (LNRT) in Mozambique in duplicate. Cultures grew in Stonebrink (ST) medium with pyruvate and Lowenstein-Jensen (LJ) medium with glycerol. DNA extraction was performed according to the protocol described by Van Soolingen and colleagues [20]. The molecular identification of *Mycobacterium tuberculosis* complex (MTBC) strains was based on the use of two sets of multiplex PCR for identifying the genus *Mycobacterium* [21] and for the discrimination of the different MTBC members [22].

Genotyping of the *M*. *bovis* samples was performed using three methodologies: spoligotyping, region of difference (RD) analysis and MIRU-VNTR, as described before [23].

Spoligotyping of each sample was determined based on the numerical combination of the presence or absence of the spacers and identified according to mbovis.org database. RD analysis, a PCR-based method to determine the presence or absence of specific regions of difference (RD) was performed to identify four *M*. *bovis* groups named Af1, Af2, Eu1 and Eu2. All samples were genotyped using the MIRU-VNTR 24 loci kit (Genoscreen, France) that includes 24 size-variation markers, although only a few have proven to be polymorphic in *M*. *bovis*. The genotypic profiles were reported as a series of 24 numbers corresponding to the number of alleles at each of the 24 loci.

### Comparative dataset

We collected the largest possible database of genotypes for *M*. *bovis* in Africa. A list of sampled locations and numbers is indicated in S1 Table and S2 Table. We included African *M*. *bovis* data that encompasses the 43 spoligotyping markers and 5 VNTR markers that were present in most studies (MIRU2165, MIRU2461, MIRU577, MIRU580 and MIRU3192 or ETR-A, -B, -C, -D and -E), that previously displayed enough resolution in the African context [23, 24]. We allowed one marker per sample to be missing, whose allele will be further extrapolated in the phylogenetic analysis below. We included a European Mediterranean dataset for comparison [25]. We also included other members from the *M*. *tuberculosis* Complex (MTBC) available at the same genotyping resolution obtained from the reference database of MIRU-VNTR Plus [26] that included genotypes from 31 *M africanum*, 2 *M*. *canetti*, 11 *M*. *caprae*, 2 *M*. *pinnipedii*, 6 *M*. *microti* and 123 *M*. *tuberculosis* strains. These genotypes were included in the phylogenetic reconstruction and the dataset of European *M*. *tuberculosis* strains were used as an outgroup for the population tree analysis of *M*. *bovis* strains.

### Phylogenetics reconstruction and statistical analyses of *M*. *bovis* genotypes

Reconstruction was performed using a matrix of the 48 markers described above and by applying the reduced median algorithm [27] followed by the median joining algorithm [28]. Both present at the network software (freely available at http://www.fluxus-engineering.com), as we suggested for MIRU-VNTR data in *M*. *tuberculosis* [29, 30]. Given the faster evolutionary rate of VNTR markers against binary spoligotyping markers we placed a greater weight of the latter in the analyses against VNTR markers (15 against 10 respectively). Network allows missing genotyping information in the input data that will be extrapolated from the parsimonious phylogenetic analysis.

The diversity of the 48 makers was converted into a binary matrix reflecting genetic distances between each genotype. That binary matrix was used to calculate diversity indices in DNAsp 6 [31]. For each individual population (country) with sample size higher than 20, we estimated the number of haplotypes *h*, the haplotype diversity *Hd* (probability that two randomly sampled haplotypes are different), the average number of differences *K* (average number of differences between all the pairs in a population) and the nucleotide diversity *Pi* (average number of differences between all the pairs in a population per number of loci).

In order to estimate the coalescence point of *M*. *bovis* in each population, we used the previously described outgroups (corresponding to genotypes of six other members of the MTBC) to define the root with Network software. The root was used to obtain the ρ statistic for each dataset. The ρ statistic measures the average number of differences from a root (coalescence point) and each of the samples and represents an unbiased model-free diversity measure and time estimator [32].

The different statistics were plotted geographically using the Kriging algorithm of Surfer 8 software using the geographic points indicated in S1 Fig. A geographically central point of each country was selected as the representative data point instead of the capital where, in many cases, was close to the border with other countries and near the capital of the other country.

*Fst* distances between pairs of populations were calculated using DNAsp 6 [31] and a population tree (using neighbour-joining) was calculated from the *Fst* matrix obtained using MEGA7 [33]. We performed the analysis with and without the locations with lower sample size (Burundi and Eritrea), excluding Uganda nevertheless (only 2 genotypes). We included a subset of European *M*. *tuberculosis* genotypes to be used as outgroup in the analysis.

## Results

To investigate the presence of hypothetical genetic reservoirs of *M*. *bovis* across Africa, we performed a detailed phylogeographic and statistical analysis on African diversity of *M*. *bovis*. For that we collected 1504 genotypes comprised by 48 markers (43 biallelic and 5 multiallelic) (S1 Table) to which we added 45 new genotypes from Mozambique.

**Fig 1:**
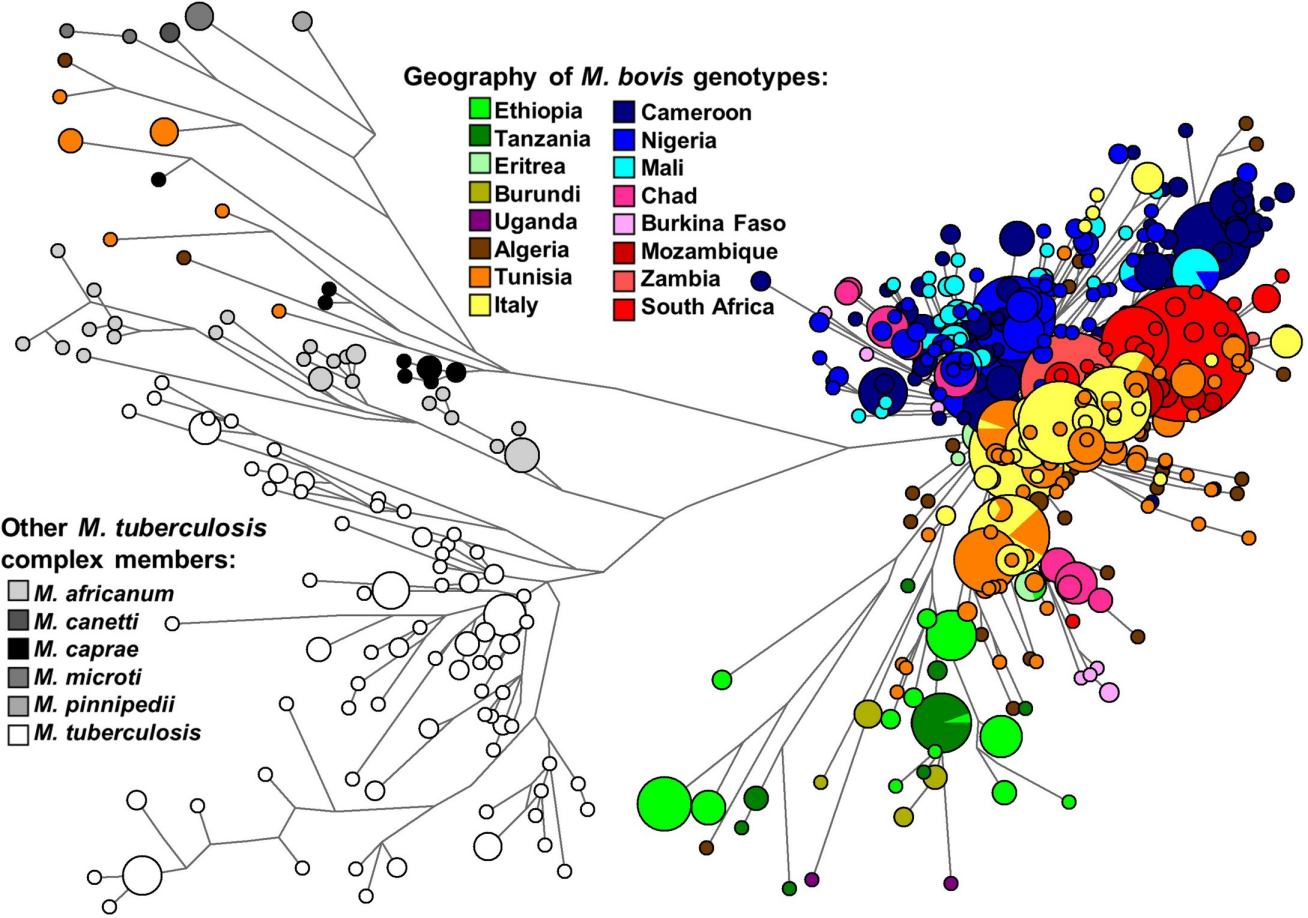
Median network displaying diversity of *Mycobacterium bovis* in Africa using 48 markers (43 spoligotype spacers and 5 VNTRs). The genotypes of *M*. *tuberculosis*, *M*. *caprae*, *M*. *africanum*, *M*. *canetti* and *M*. *pinnipedii* strains were included as outgroups. Samples were coloured according to the geographic location (in the case of *M*. *bovis* strains) or according to the strains (in the case of other *M*. *tuberculosis* complex strains). Figure was made using freely available phylogenetic software network (http://www.fluxus-engineering.com).

We reconstructed a phylogeny for the genotypes of *M*. *bovis* strains in Africa (Fig 1). Incidentally, some strains from Northern Africa clustered with *M*. *caprae* instead of *M*. *bovis*. The presence of other MTBC strains in the network shows a deep coalescence point on the convergence of *M africanum*, *M*. *canetti*, *M*. *caprae*, *M*. *pinnipedii*, *M*. *microti* and *M*. *tuberculosis* genotypes and *M*. *bovis* strains’ genotypes. This allows to identify branches that are coalescing at a deeper evolutionary point than most of the *M*. *bovis* lineages. These ancient lineages are mostly from Eastern Africa (Ethiopia, Tanzania) and Northern Africa (Algeria, Tunisia), although some haplotypes with minor frequencies from Central Africa are also present. On an overview analysis of the network, Eastern and Northern Africa seem to display a deeper ancestry of *M*. *bovis* compared with other African regions.

Most of the *M*. *bovis* genotypes radiate into several clades from a single major coalescence point. On that coalescence point it is possible to discern a few longer branches from Ethiopia and Tanzania. There are specific branches from Northern Africa, Mozambique, Southern Africa, Italy and Central Africa, reflecting a strong geographical compartmentalization. Most of the diversity in Central Africa seems present on a single major clade. That clade, defined by the spacer 30, corresponds likely to the common African spoligotype type Af1. Mozambique presents three clades from the main *M*. *bovis* radiation point, including one shared with South Africa, but, in general, Mozambique displays, as other locations, a specific local subset of diversity.

In general, the sharing of genotypes across borders is mostly restricted to countries in the vicinity of each other. However, Northern Africa (Algeria) shows common haplotypes with South Africa (Fig 1 and S3 Table). Some of the matching haplotypes could be defined as previously named European clusters Eu1 and Eu2 since they lack spacers 11 and 21 respectively in the spoligotyping data that has have been used to define these clusters [23, 34]. However, spoligotyping data also shows a great level of homoplasy and these definitions might not be exact. It is interesting to notice that, as somewhat expected, there is a great level of sharing between North Africa and the sample from Italy. High levels of gene flow and trading are visible from pre-history until recent times between both regions [35].

**Fig 2:**
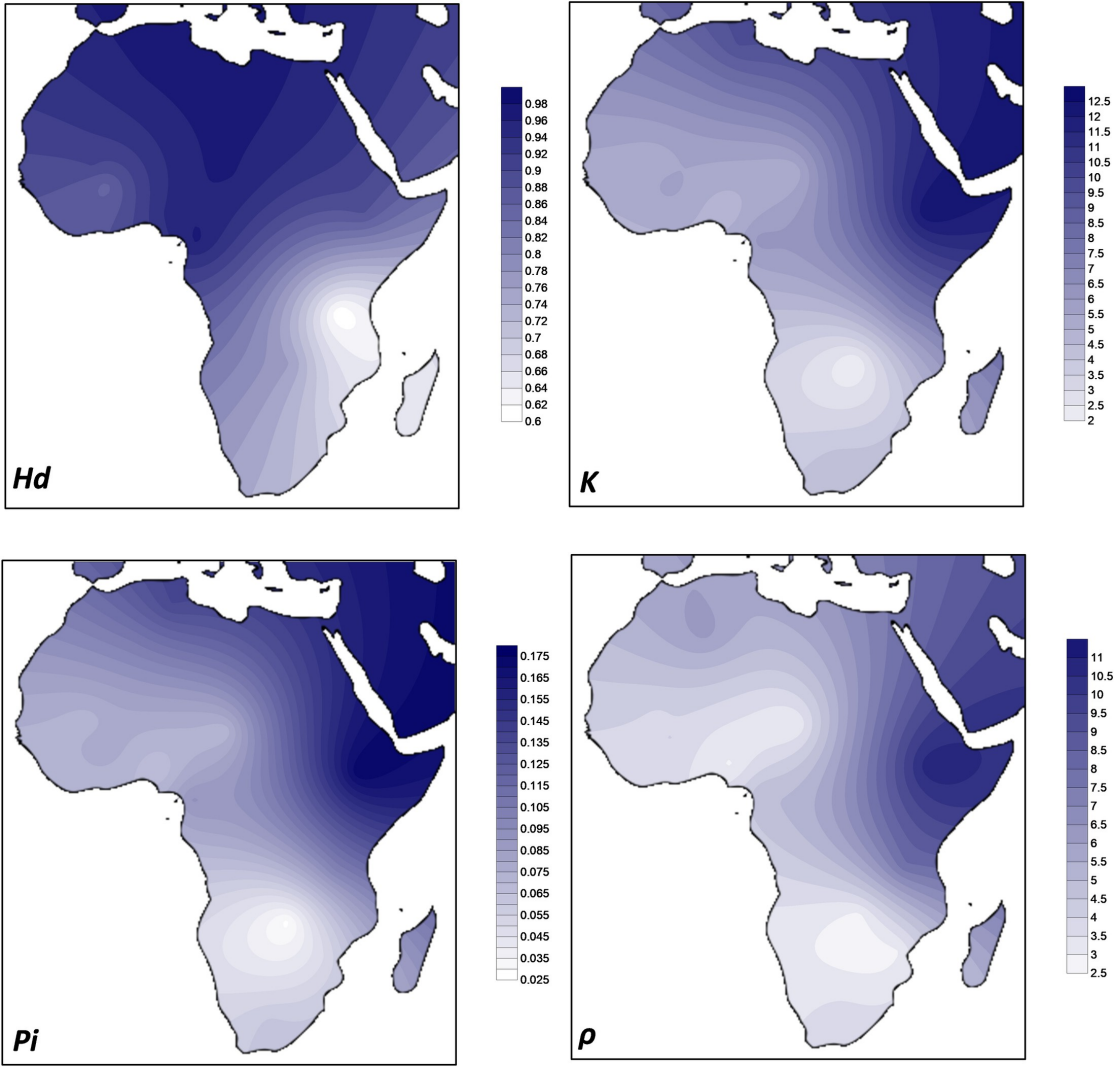
Geographic distribution of diversity of *Mycobacterium bovis* in Africa using the Kriging algorithm of Surfer 8 software for four diversity estimators (haplotype diversity *Hd*, the average number of differences *K*, the nucleotide diversity *Pi* and *ρ*). Map outline was adapted from https://commons.wikimedia.org/wiki/Atlas_of_the_world.

In order to visualize the distribution of the genetic diversity of *M*. *bovis* in Africa, we obtained four diversity statistics in all populations with more than 20 samples (Table 1) that were plotted geographically (Fig 2). The measures have different properties. Haplotype diversity *Hd* reflects the high or low number and frequency of circulating haplotypes independently of the distance between the haplotypes. Recently established reservoirs of diversity would be best detected with this measure than the others. *ρ* measures depth of diversity in a phylogenetic context and it could establish deep or shallow presence of *M*. *bovis* in each region even if concentrated in a small number of haplotypes. *K* and *Pi* offers intermediate properties (similar haplotypes have zero distance between them but further genetic distances between other pairs of haplotypes are also accounted).

**Table 1:** Diversity measures obtained from the sample of *Mycobacterium bovis* genotypes in different countries. Diversity indices were not calculated for locations with low sample size (n<20).

Country	Sample size (n)	Number of haplotypes, *h*	Haplotype diversity, *Hd*	Average number of differences, *K*	Nucleotide diversity, *Pi*	*Rho*, *ρ*
**Algeria**	89	52	0.95812	7.56844	0.10660	6.2410
**Burkina Faso**	32	11	0.85238	5.73333	0.08075	4.0313
**Burundi**	10	4	-	-	-	-
**Cameroon**	269	84	0.96288	6.18543	0.09120	4.9776
**Chad**	67	34	0.96201	5.07825	0.07152	3.0000
**Eritrea**	14	5	-	-	-	-
**Ethiopia**	67	15	0.88105	12.37268	0.17426	10.881
**Mali**	59	11	0.90181	5.04734	0.07109	3.8644
**Mozambique**	102	14	0.67618	3.62667	0.05108	2.9314
**Nigeria**	178	68	0.94160	4.65454	0.06556	2.9270
**South Africa**	193	26	0.73343	4.38957	0.06182	3.8083
**Tanzania**	27	8	0.60114	7.03704	0.09911	7.9259
**Tunisia**	211	89	0.97319	9.54557	0.13499	5.5707
**Uganda**	2	2	-	-	-	-
**Zambia**	24	6	0.75725	2.00725	0.02827	2.8333
**Italy**	205	33	0.87972	4.66361	0.06565	3.0878

It is clear that countries in Eastern Africa (Ethiopia and Tanzania) display the higher values of diversity when measures related with deep ancestry are concerned (*ρ*, *K*, *Pi*), mainly in the case of *ρ*, followed by Northern Africa (Algeria and Tunisia) that display very high values in terms of *K* and *Pi*, comparable to Eastern Africa indices. Countries in Central/Western Africa display lower diversity but in general countries in Southern Africa (Mozambique, Zambia and South Africa) display the lowest values. A gradient from North to South is visible in in the maps (Fig 2). It is important to point out that this gradient is disrupted when moving North from Africa. The diversity in the Mediterranean Europe sample is substantially lower than in North Africa, reinforcing the status of Northern and Eastern Africa as regions of deep ancestry of *M*. *bovis* diversity.

In terms of *Hd*, a statistic that could better reflect number of circulating strains, Northern and Central Africa display the highest values. In contrast, neighbour-countries like Mozambique, Tanzania and Zambia display the lowest diversity in terms of this statistics.

**Fig 3:**
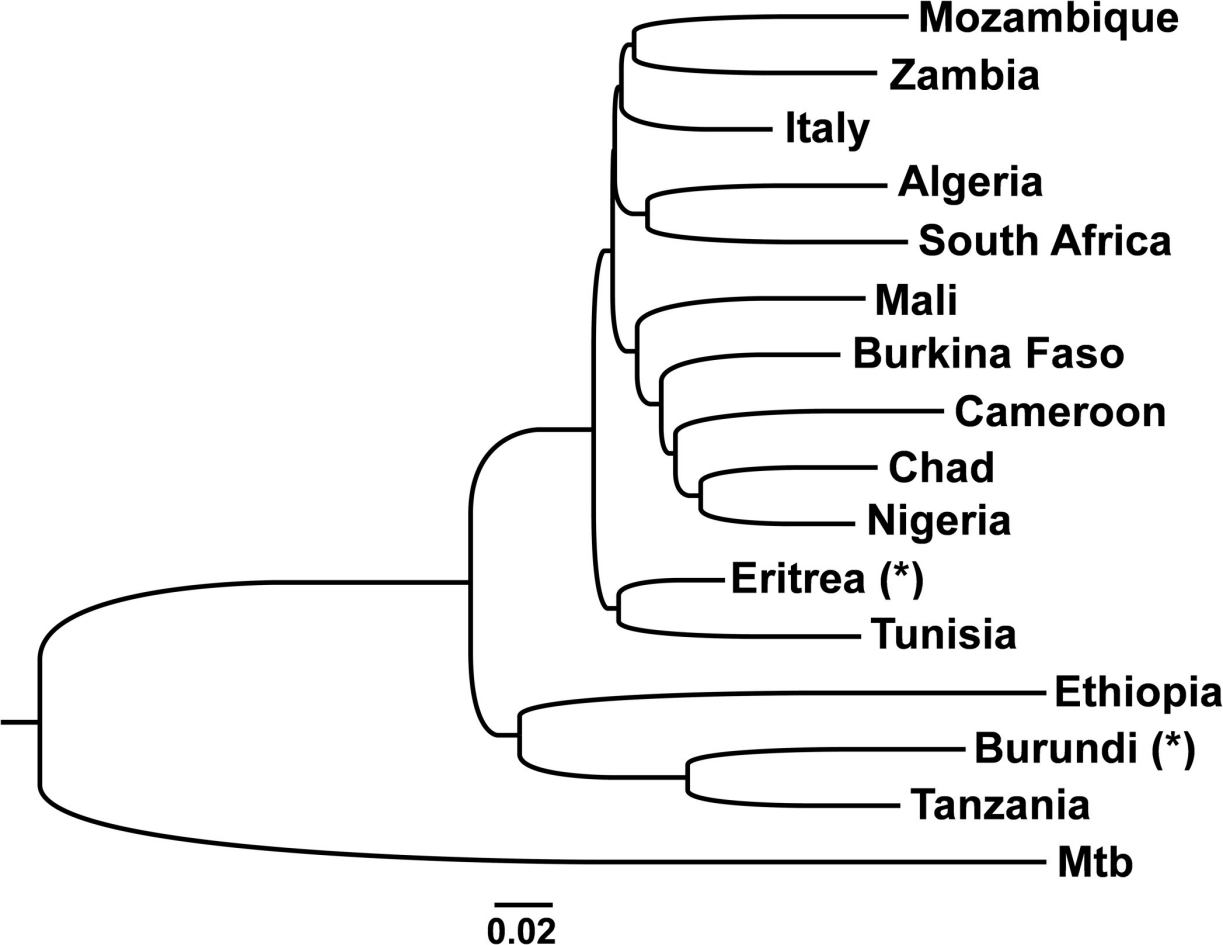
Neighbour-joining population tree based on a matrix of *Fst* values between populations. A dataset of European *M*. *tuberculosis* (Mtb) was used as an outgroup. Sample sets indicated as (*) have a low sample size and their placement in the tree is possibly uncertain.

In order to fully evaluate the relationships between countries in terms of general diversity we calculated a population neighbour-joining tree based on *Fst* values. In one analysis, we included the low sample size locations Burundi and Eritrea in order to check if they fit the general patterns. Their presence did not alter the obtained relationship between the other groups so we present that analysis. The results clearly show Ethiopia and Tanzania as the most divergent populations, branching earlier in the tree (Fig 3). Burundi, on the limit between Eastern and Central Africa clusters with Eastern Africa, despite its low sample size. These were followed by the clustered Eastern Africa Eritrea and North African Tunisia, while the other Northern African population, Algeria, due to the sharing of haplotypes with South Africa was clustered with that population. Southern African Mozambique and Zambia were also clustered. The Italian population is marginally clustered with Mozambique and Zambia but essentially forms a cluster with Algeria and the three Southern African countries in contrast with countries in Central and Western Africa that were also grouped in a separate cluster. Nevertheless, diversity in Central/western Africa and Southern Africa seem more restricted than Eastern and Northern African diversity. In general, the population tree supports the previously performed analyses in terms of phylogeography and general statistics.

**Fig 4:**
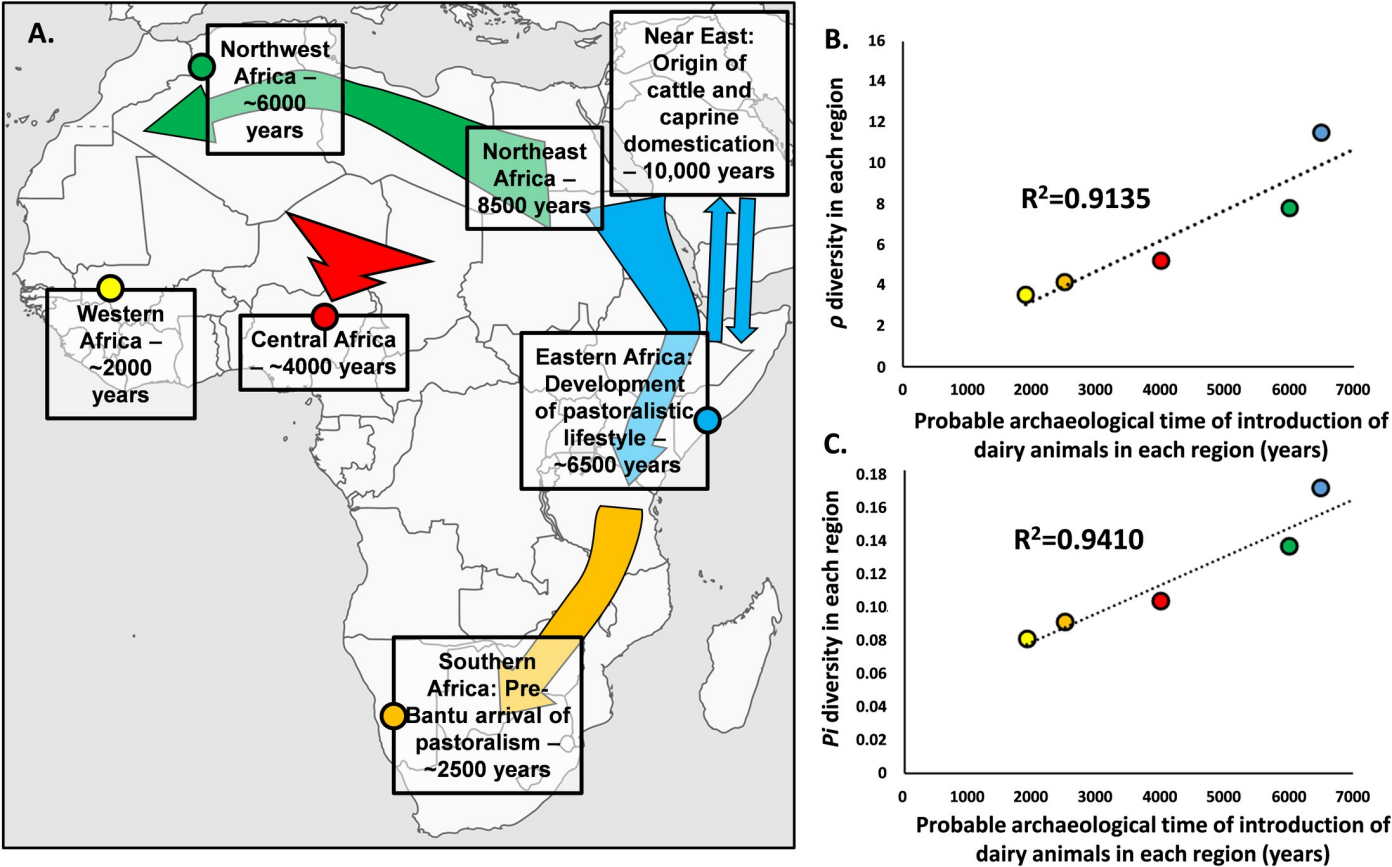
Comparison between hypothetical time of introduction of dairy animals across Africa from the archaeological record (A.) and genetic diversity statistics, *ρ* (B.) and *Pi* (C.). Colour of data points in (B) and (C) correspond to the regions of the same colour in the map (A). Map was adapted from https://commons.wikimedia.org/wiki/Atlas_of_the_world.

In order to establish general values per region we calculated *ρ* and *Pi* for the different general regions and, expectedly, for both measures Eastern Africa was the most diverse (*ρ* = 11.611, *Pi* = 0.17113), followed by Northern Africa (*ρ* = 7.8564, *Pi* = 0.136349), Central Africa (*ρ* = 5.3448, *Pi* = 0.10376), Southern Africa (*ρ* = 4.2922, *Pi* = 0.09087) and Western Africa (ρ = 3.978, Pi = 0.08087) (Fig 4).

## Discussion

*M*. *bovis* is the main organism responsible for bTB, a form of TB present in cattle and other domestic and wildlife species, that can be transmitted to humans through zoonosis. bTB represents a high burden in the African continent. In order to understand how the genetic diversity of this pathogen is distributed across Africa, we performed a genetic analysis on its overall diversity. This allows to establish probable regions that can be genetic reservoirs in the continent and where public health actions should focus aiming at bTB eradication. There are two conclusions that erupt from this analysis:

A) One feature is that the transportation of the pathogen across borders is probably more limited than expected–each country displays a subset of diversity that might have been established and maintained for a long period; except for the case of South Africa and Algeria, shared haplotypes seems to occur almost exclusively between neighbour countries. The South African/Algeria sharing of haplotypes can be related with an outside African source. Some of the diversity within these two countries are likely from clades Eu1 and these represent the matches detected, possibly reflecting *M*. *bovis* introduced by Europeans in South Africa and Algeria.

B) Considering the haplotype diversity, the number of circulating strains in different regions differs considerably across Africa. In the present analysis Mozambique, Tanzania and Zambia display the lowest diversity in circulating strains and are minor reservoirs of genetic diversity of *M*. *bovis* which typically represent more simplistic scenarios for public health than larger genetic reservoirs. However, a real scenario can just be attained by obtaining reliable values of incidence of bTB in different African countries that can be combined with this information. While Mozambique, Tanzania and Zambia show little diversity of circulation genotypes, their estimate of cases that could be related with recent transmission from genetic data is over 70% in the three countries, highlighting probable recent outbreaks.

Nevertheless, the distribution of the diversity of *M*. *bovis* in Africa raises questions regarding the processes that shaped that diversity. There has been a wide discussion regarding the origin and spread of the *Mycobacterium tuberculosis* complex that includes *M*. *tuberculosis*, the infectious agent responsible for TB, and *M*. *bovis*, the causal infectious agent of bTB that mostly infects animals but that is transmitted to humans. One common hypothesis for the origin of the MTBC relates with the emergence of agriculture in the Fertile Crescent in the Neolithic period, over 10,000 years ago [36], when cattle and other animal were domesticated. While, in the past, an animal-to-human transmission hypothesis was in place for the emergence of *M*. *tuberculosis* from *M*. *bovis* the sequencing of both genomes clearly showed that the pathogens are not directly related [37] but instead they share a common ancestor. In fact, several studies indicate that the diversity of *M*. *tuberculosis* suggests a coalescence of the organism above 40,000 years ago or above, linking its global spread to a probable co-expansion with the modern humans Out-of-Africa [15, 38]. On the other hand, estimates of the coalescence time of *M*. *bovis* are as recent as 6000 years placing it well within the Holocene and potentially with the expansion of Neolithic or pastoralist societies in Africa.

It is possible that bTB arose when animals were kept in larger groups in limited areas, that allowed the disease to be more easily transmitted between individuals. Domestication in Africa occurred through introductions from the Near East, where cattle was domesticated about 10,000 years ago, marking also the initiation of dairy practices. None of the native African animals were domesticated. In that sense we can hypothesize that bTB has a Neolithic origin and it was spread across Africa concomitantly with the introduction of cattle. Domestication of cattle and the maintenance of larger groups of animals is also connected with the use of animals, not only as a source of meat and hides, but also as milk producers, part of the so-called Secondary Product Revolution. That change in societies goes beyond a cultural change since it has deep biological implications, as part of the populations was positively selected through time in order to be able to digest lactose in adulthood. Incidentally, the ability to consume milk facilitates zoonotic transfers as it is clear nowadays that milk consumption is the major mode of transmission of bTB to humans. This suggests a co-evolution of humans, cattle and pathogen that allowed the endurance of the disease. Could the data suggest that these genetic patterns are related with anciently established routes? Two points of evidence support this scenario. The first is the strong geographic clustering of the diversity across Africa that provides a direct evidence that this diversity was established in the past and enough time elapsed for local diversity to be established. The second is that the levels of diversity across Africa, that could illustrate different timeframes for diversity to accumulate in each region, correlate well with the estimated arrival of domesticated cattle and pastoralism into those regions (Fig 4).

The two major areas with higher diversity in Africa are Eastern Africa and Northern Africa. These were the first analysed areas where domesticated cattle, goats and sheep were introduced from the Near East [16]. Following an earlier spread of these domesticates into Northeast Africa by 8000 years [16], Eastern Africa, with the higher diversity in our analysis, saw an introduction of people, animals and domestication practices possibly as early as 6500–6000 years ago where a nomadic pastoralistic lifestyle emerged. Specific alleles for lactase persistence, a trait that indicates human co-evolution with cattle, sheep and goat and milk consumption in adulthood, developed and were positively selected in Eastern Africa [19], including the major allele C-14010, dated to around 6–7000 years [19], the time of introduction of domesticated animals. Recent research on ancient DNA suggests a clear admixture of early pastoralistic groups with Eastern Africans by at least 4000 years [39]. Nevertheless, Eastern Africa also display a long history of contact with Arabia/Near East [40, 41] that could have promote multiple transmission events of domesticates and pathogen further increasing the diversity of the *M*. *bovis* in Eastern Africa.

As mentioned above, North Africa saw an earlier introduction to the area that is nowadays Egypt (8000 years ago), however in Northwestern Africa (location of our samples) pastoralism was introduced about 6000 years ago [42, 43]. The Sahel Belt was a major travelling corridor both on a West/East axis or North/South [44], where nomadic pastoralistic groups moved. The increased aridity of the Sahara caused pastoralistic groups to migrate South in the Late Holocene. By 4000 years ago, pastoralistic societies and herding practices were archaeologically detected in Nigeria [45] and by 3000 years in Cameroon [46]. Sub-Saharan Western Africa displays low diversity of *M*. *bovis* genotypes. While some earlier cases of cattle domestication might exist in this area [47, 48], clear signals of introduction of modern cattle species dates to first millennium AD in Burkina Faso [49].

Populations in Southern Africa, before the massive expansion of Bantu-speaking agriculturists in the last two millennia from Central Africa [50] that replaced part of the indigenous populations, were mostly isolated from the remaining continent [51, 52]. However, frequencies of the previously mentioned lactase persistence allele C-14010 in the indigenous KhoeSan groups suggests a migration that carried that allele, hardly present in Bantu-speaking populations from the South. Such migration probably introduced domesticated species and pastoralistic practices into Southern Africa about 2500 years ago [18], further supported by archaeology [53].

The probable time of introduction of domesticated animals across the continent (Fig 4A) is reflected in the order of diversity values for the *ρ* and *Pi*, the statistics that better reflect time and depth of accumulated diversity. More exactly, both set of statistics (Fig 4B and Fig 4C, respectively) correlate well with the hypothetical time of introduction of cattle, sheep and goat. That correlation would be even stronger (>90%) if we consider that the diversity of Eastern Africa could have had later additions from the Near East/Arabia Peninsula, the hypothetical place of origin for *M*. *bovis*. In conclusion, using a phylogeographic and population genetics approach, we suggest that the current patterns of diversity of *M*. *bovis* in Africa were established in prehistoric times, with the pathogen being carried by introduced domesticated animals across the continent.

It is interesting to highlight that the diversity in Mediterranean Europe was substantially lower than in Mediterranean Africa. If the coalescence estimate for *M*. *bovis* is actually below 8000 years [15] the pathogen would not have been carried by the main Neolithic wave from the Near East into Europe, and the Mediterranean low diversity observed could have the result of gene flow from Northern Africa to Southern Europe as observed in human genetics [35, 54].

Spoligotyping and MIRU-VNTR data offer a useful insight into the diversity and transmission of *M*. *bovis* and a clear model for the establishment of *M*. *bovis* genetic structure in Africa. The current study is limited by the sampling with many locations across Africa without genotyping data. The analysis performed here offers a parsimonious model based on the available data extrapolated for the overall continent. This model can be further tested with more data. In the future, complete genomes of the pathogen across Africa can provide further details to the established scenario, and importantly, it can allow to test the depth of the diversity using a molecular clock and a refined phylogenetic structure.

Understanding the nature of the diversity is of great importance and could dictate the plan of action of public health agents. Strategies should be adjusted for either recently established diversity, possible due to economic trades with other countries, whose public health actions should focus on control of imports and early diagnosis of infected animals; while long–term established diversity can prove more difficult to tackle. In the case where pathogen diversity could have been established for thousands of years, as proposed here, strains could be strongly adapted to local environment and hosts, evolving through strong co-evolution with the domesticates, potentially with wild animals and, zoonotically, with humans.

## Supporting information

S1 Fig African map displaying the geographic datapoints for *Mycobacterium bovis* genotypes used in the phylogeographic analysis. The colours of the different points correspond to the network colour code in Fig 1. Map outline was adapted from https://commons.wikimedia.org/wiki/Atlas_of_the_world.
(PDF)

S1 Table *Mycobacterium bovis* samples used in the phylogeographic and population genetics analyses, with the references from where data was obtained. (PDF)

S2 Table Characteristics of *Mycobacterium bovis* genotypes, including sample identification, location of collection, MIRU-VNTR genotype code and spoligotype, from the new samples for Mozambique. (PDF)

S3 Table Percentage of genotypes from a specific African country (columns) that are shared with other African countries (lines). (PDF)
