# Supplementary material for: Evolutionary analysis of Mycobacterium bovis genotypes across Africa suggests co-evolution with livestock and humans
Source: PLoS Negl Trop Dis. 2020 Mar 2;14(3):e0008081. doi: 10.1371/journal.pntd.0008081 (PMC7077849; doi:10.1371/journal.pntd.0008081)
Supplement: S1 Fig — The colours of the different points correspond to the network colour code in Fig 1. Map outline was adapted from https://commons.wikimedia.org/wiki/Atlas_of_the_world. (PDF) [file pntd.0008081.s001.pdf]

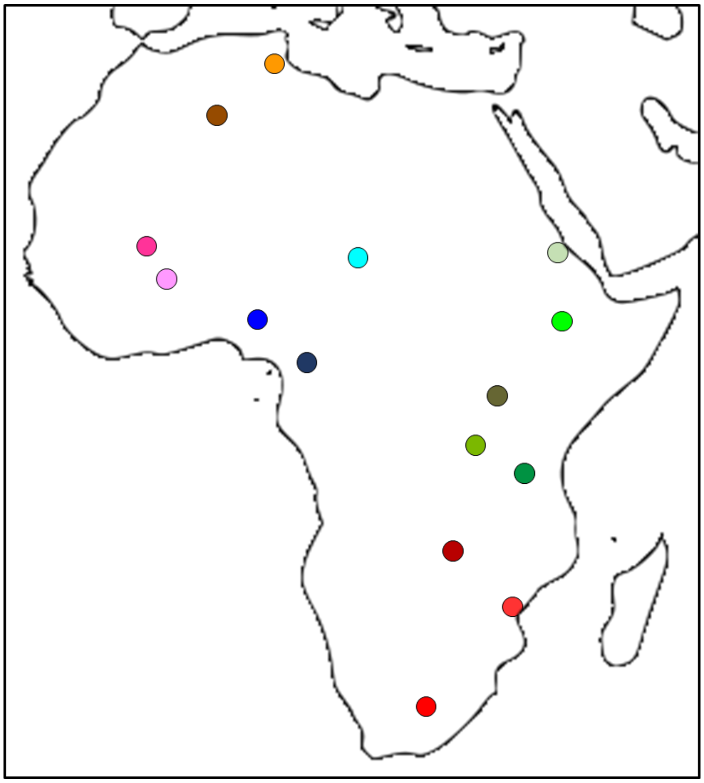

S1 Fig. African map displaying the geographic datapoints for *Mycobacterium bovis* genotypes used in the phylogeographic analysis. The colours of the different points correspond to the network colour code in Figure 1. Map outline was adapted from https://commons.wikimedia.org/wiki/Atlas_of_the_world.
