# Supplementary material for: Evolutionary analysis of Mycobacterium bovis genotypes across Africa suggests co-evolution with livestock and humans
Source: PLoS Negl Trop Dis. 2020 Mar 2;14(3):e0008081. doi: 10.1371/journal.pntd.0008081 (PMC7077849; doi:10.1371/journal.pntd.0008081)
Supplement: S1 Table — (PDF) [file pntd.0008081.s002.pdf]

S1 Table. ***Mycobacterium bovis*** **samples used in the phylogeographic and population genetics analyses, with the references from where data was obtained.**

Country	Region	Sample number	Reference
Algeria	North Africa	89	[1]
Burkina Faso	Western Africa	32	[2]
Burundi	Eastern Africa	10	[3]
Cameroon	Central Africa	269	[4]<br>[3]<br>[5]
Chad	Central Africa	67	[4]<br>[3]
Eritrea	Eastern Africa	14	[6]
Ethiopia	Eastern Africa	67	[3]<br>[7]
Mali	Western Africa	59	[8]<br>[4]<br>[3]
Mozambique	Southern Africa	102	[9]<br>*Unpublished, this work*
Nigeria	Central Africa	178	[4]<br>[3]<br>[10]
South Africa	Southern Africa	193	[11]<br>[12]<br>[13]
Tanzania	Eastern Africa	27	[4]<br>[3]<br>[14]
Tunisia	North Africa	211	[15]<br>[16]<br>[17]
Uganda	Central Africa	2	[3]
Zambia	Southern Africa	24	[18]
Italy	Europe	205	[19]

References

1. Sahraoui N, Müller B, Guetarni D, Boulahbal F, Yala D, Ouzrout R, et al. Molecular characterization of *Mycobacterium bovis* strains isolated from cattle slaughtered at two abattoirs in Algeria. BMC Veterinary Research. 2009;5:4-. doi: 10.1186/1746-6148-5-4. PubMed PMID: PMC2640374.
2. Sanou A, Tarnagda Z, Kanyala E, Zingué D, Nouctara M, Ganamé Z, et al. *Mycobacterium bovis* in Burkina Faso: Epidemiologic and Genetic Links between Human and Cattle Isolates. PLOS Neglected Tropical Diseases. 2014;8(10):e3142. doi: 10.1371/journal.pntd.0003142.
3. Berg S, Garcia-Pelayo MC, Müller B, Hailu E, Asiimwe B, Kremer K, et al. African 2, a Clonal Complex of *Mycobacterium bovis* Epidemiologically Important in East Africa. Journal of Bacteriology. 2011;193(3):670-8. doi: 10.1128/jb.00750-10.
4. Müller B, Hilty M, Berg S, Garcia-Pelayo MC, Dale J, Boschiroli ML, et al. African 1, an Epidemiologically Important Clonal Complex of *Mycobacterium bovis* Dominant in Mali, Nigeria, Cameroon, and Chad. Journal of Bacteriology. 2009;191(6):1951-60. doi: 10.1128/jb.01590-08.

5. Egbe NF, Muwonge A, Ndip L, Kelly RF, Sander M, Tanya V, et al. Molecular epidemiology of *Mycobacterium bovis* in Cameroon. Scientific Reports. 2017;7(1):4652. doi: 10.1038/s41598-017-04230-6.
6. Ghebremariam MK, Hlokwe T, Rutten VPMG, Allepuz A, Cadmus S, Muwonge A, et al. Genetic profiling of Mycobacterium bovis strains from slaughtered cattle in Eritrea. PLOS Neglected Tropical Diseases. 2018;12(4):e0006406. doi: 10.1371/journal.pntd.0006406.
7. Firdessa R, Tschopp R, Wubete A, Sombo M, Hailu E, Erenso G, et al. High Prevalence of Bovine Tuberculosis in Dairy Cattle in Central Ethiopia: Implications for the Dairy Industry and Public Health. PLOS ONE. 2012;7(12):e52851. doi: 10.1371/journal.pone.0052851.
8. Müller B, Steiner B, Bonfoh B, Fané A, Smith NH, Zinsstag J. Molecular characterisation of *Mycobacterium bovis* isolated from cattle slaughtered at the Bamako abattoir in Mali. BMC Veterinary Research. 2008;4:26-. doi: 10.1186/1746-6148-4-26. PubMed PMID: PMC2483712.
9. Machado A, Rito T, Ghebremichael S, Muhate N, Maxhuza G, Macuamule C, et al. Genetic diversity and potential routes of transmission of Mycobacterium bovis in Mozambique. PLOS Neglected Tropical Diseases. 2018;12(1):e0006147. doi: 10.1371/journal.pntd.0006147.
10. Jenkins AO, Cadmus SIB, Venter EH, Pourcel C, Hauk Y, Vergnaud G, et al. Molecular epidemiology of human and animal tuberculosis in Ibadan, Southwestern Nigeria. Veterinary Microbiology. 2011;151(1):139-47. doi: http://dx.doi.org/10.1016/j.vetmic.2011.02.037.
11. Hlokwe TM, Jenkins AO, Streicher EM, Venter EH, Cooper D, Godfroid J, et al. Molecular characterisation of *Mycobacterium bovis* isolated from African buffaloes (*Syncerus caffer*) in Hluhluwe-iMfolozi Park in KwaZulu-Natal, South Africa2011.
12. Hlokwe TM, van Helden P, Michel A. Evaluation of the Discriminatory Power of Variable Number of Tandem Repeat Typing of *Mycobacterium bovis* Isolates from Southern Africa. Transboundary and Emerging Diseases. 2013;60:111-20. doi: 10.1111/tbed.12096.
13. Jolly M, Tiny H, Tanguy M, Ben JAdP, Anita LM. Spillover of *Mycobacterium bovis* from Wildlife to Livestock, South Africa. Emerging Infectious Disease journal. 2015;21(3):448. doi: 10.3201/eid2103.131690.
14. Katale BZ, Mbugi EV, Siame KK, Keyyu JD, Kendall S, Kazwala RR, et al. Isolation and Potential for Transmission of *Mycobacterium bovis* at Human–livestock–wildlife Interface of the Serengeti Ecosystem, Northern Tanzania. Transboundary and Emerging Diseases. 2017;64(3):815-25. doi: 10.1111/tbed.12445.
15. Ben KI, Boschiroli ML, Souissi F, Cherif N, Benzarti M, Boukadida J, et al. Isolation and molecular characterisation of *Mycobacterium bovis* from raw milk in Tunisia. African Health Sciences. 2011;11(Suppl 1):S2-S5. PubMed PMID: PMC3220130.
16. Djemal SE, Siala M, Smaoui S, Kammoun S, Marouane C, Bezos J, et al. Genetic diversity assessment of Tunisian Mycobacterium bovis population isolated from cattle. BMC veterinary research. 2017;13(1):393-. doi: 10.1186/s12917-017-1314-y. PubMed PMID: 29246228.
17. Siala M, Cassan C, Smaoui S, Kammoun S, Marouane C, Godreuil S, et al. A first insight into genetic diversity of Mycobacterium bovis isolated from extrapulmonary tuberculosis patients in South Tunisia assessed by spoligotyping and MIRU VNTR. PLOS Neglected Tropical Diseases. 2019;13(9):e0007707. doi: 10.1371/journal.pntd.0007707.
18. Malama S, Johansen TB, Muma JB, Munyeme M, Mbulo G, Muwonge A, et al. Characterization of *Mycobacterium bovis* from Humans and Cattle in Namwala District, Zambia. Veterinary Medicine International. 2014;2014:7. doi: 10.1155/2014/187842.
19. Marianelli C, Amato B, Boniotti MB, Vitale M, Pruiti Ciarello F, Pacciarini ML, et al. Genotype diversity and distribution of Mycobacterium bovis from livestock in a small, high-risk area in northeastern Sicily, Italy. PLOS Neglected Tropical Diseases. 2019;13(7):e0007546. doi: 10.1371/journal.pntd.0007546.
