# Supplementary material for: Evolutionary analysis of Mycobacterium bovis genotypes across Africa suggests co-evolution with livestock and humans
Source: PLoS Negl Trop Dis. 2020 Mar 2;14(3):e0008081. doi: 10.1371/journal.pntd.0008081 (PMC7077849; doi:10.1371/journal.pntd.0008081)
Supplement: S2 Table — (PDF) [file pntd.0008081.s003.pdf]

S2 Table. Characteristics of *Mycobacterium bovis* genotypes, including sample identification, location of collection, MIRU-VNTR genotype code and spoligotype, from the new samples for Mozambique.

Sample ID	Subregion	ETR-A 2165	ETR-B 2461	ETR-C 577	ETR-D 580	ETR-E 3192	Spoligotype
MMP19	Namaacha	7	5	5	3	3	SB0140
MIN21	Govuro	5	5	5	3	3	SB0961
MSO10	Machanga	n	5	5	3	3	SB0961
MMP20	Machava	7	5	5	3	3	SB0140
MMA10	Gondola	7	5	5	3	3	SB0961
MMA11	Gondola	7	5	5	3	3	SB0961
MMP21	Machava	7	5	5	3	3	SB0140
MSO11	Machanga	7	5	5	3	3	SB0961
MSO12	Machanga	7	5	5	3	3	SB0961
MSO13	Machanga	n	5	5	3	3	SB2306
MIN22	Zavala	9	5	5	3	3	SB0140
MSO14	Machanga	7	5	5	3	3	SB0140
MGA2	Chibuto	6	4	5	2	3	SB2481
MGA3	Chibuto	7	5	5	3	3	SB0961
MMA12	Gondola	7	5	5	3	3	SB0961
MMP22	Machava	7	5	5	3	3	SB0140
MMP23	Chobela	n	5	5	3	3	SB0140
MMA13	Gondola	7	5	5	3	3	SB0961
MMA14	Gondola	7	5	5	3	3	SB0961
MMA15	Gondola	7	5	5	3	3	SB0961
MMA16	Gondola	7	5	5	3	3	SB0961
MMA17	Gondola	7	5	5	3	3	SB0961
MMP24	Machava	7	5	5	3	3	SB0140
MIN23	Mabote	n	5	n	3	3	SB0961
MMP25	Magude	7	5	5	3	3	SB0140
MSO15	Nhamatanda	6	5	5	3	3	SB0140
MMA18	Gondola	7	5	5	3	3	SB0961
MMA19	Gondola	7	5	5	3	3	SB0961
MMA20	Gondola	7	5	5	3	3	SB0961
MMA21	Gondola	7	5	5	3	3	SB0961
MGA4	Chicumbane	6	4	5	2	3	SB2481
MSO16	Machanga	8	5	5	3	3	SB1099
MMP26	Matutuine	7	5	5	3	3	SB0961
MSO17	Machanga	7	5	n	3	3	SB2306
MSO18	Machanga	n	5	5	3	3	SB0961
MSO19	Machanga	7	5	5	3	3	SB2306
MMP27	Namaacha	8	5	5	3	3	SB0961
MMA22	Gondola	7	5	5	3	3	SB0961
MMA23	Gondola	7	5	5	3	3	SB0961
MGA5	Chicumbane	6	4	5	2	3	SB2481
MIN24	Govuro	n	5	5	3	n	SB0961
MIN25	Govuro	n	5	5	3	3	SB0961
MIN26	Govuro	7	5	5	3	3	SB0961
MMA24	Gondola	7	5	5	3	3	SB0961
MIN27	Govuro	7	5	n	3	3	SB0961
