# Supplementary material for: Evolutionary analysis of Mycobacterium bovis genotypes across Africa suggests co-evolution with livestock and humans
Source: PLoS Negl Trop Dis. 2020 Mar 2;14(3):e0008081. doi: 10.1371/journal.pntd.0008081 (PMC7077849; doi:10.1371/journal.pntd.0008081)
Supplement: S3 Table — (PDF) [file pntd.0008081.s004.pdf]

S3 Table. Percentage of genotypes from a specific African country (columns) that are shared with other African countries (lines).

Population	Algeria	Tunisia	Eritrea	Ethiopia	Tanzania	Uganda	Burundi	Mali	Cameroon	Nigeria	Burkina Faso	Chad	Zambia	Mozambique	South Africa	Percentage of haplotypic matches (%) with:
N	89	102	14	67	27	2	10	59	269	178	21	67	24	102	193	
		3.8	57.1												18.1	Algeria
	10.1		7.1													Tunisia
	3.4	3.8		6.0												Eritrea
			28.6		62.7											Ethiopia
				1.5												Tanzania
																Uganda
																Burundi
												4.5				Mali
										33.1	71.4	13.4				Cameroon
									3.0			40.3				Nigeria
									5.9							Burkina Faso
								5.1	3.0	29.2						Chad
																Zambia
																Mozambique
	19.1															South Africa
